# Supplementary material for: Predicting visual function by interpreting a neuronal wiring diagram
Source: Nature. 2024 Oct 2;634(8032):113–23. doi: 10.1038/s41586-024-07953-5 (PMC11446822; doi:10.1038/s41586-024-07953-5)
Supplement: Supplementary file 6 — CRF and ERF predictions for individual TmY4 and TmY9 cells. Analogous to Supplementary Data 3, but for TmY target types. Shown are the top four monosynaptic pathways, the strongest pathway passing through each of the top ten intermediary types (ranking from Extended Data Fig. 7), and the trisynaptic pathway Tm1–TmY–Dm3–TmY (see the section entitled Prediction of spatial normalization). [file 41586_2024_7953_MOESM6_ESM.zip › DataS4/TmY9q/720575940624808171.pdf]

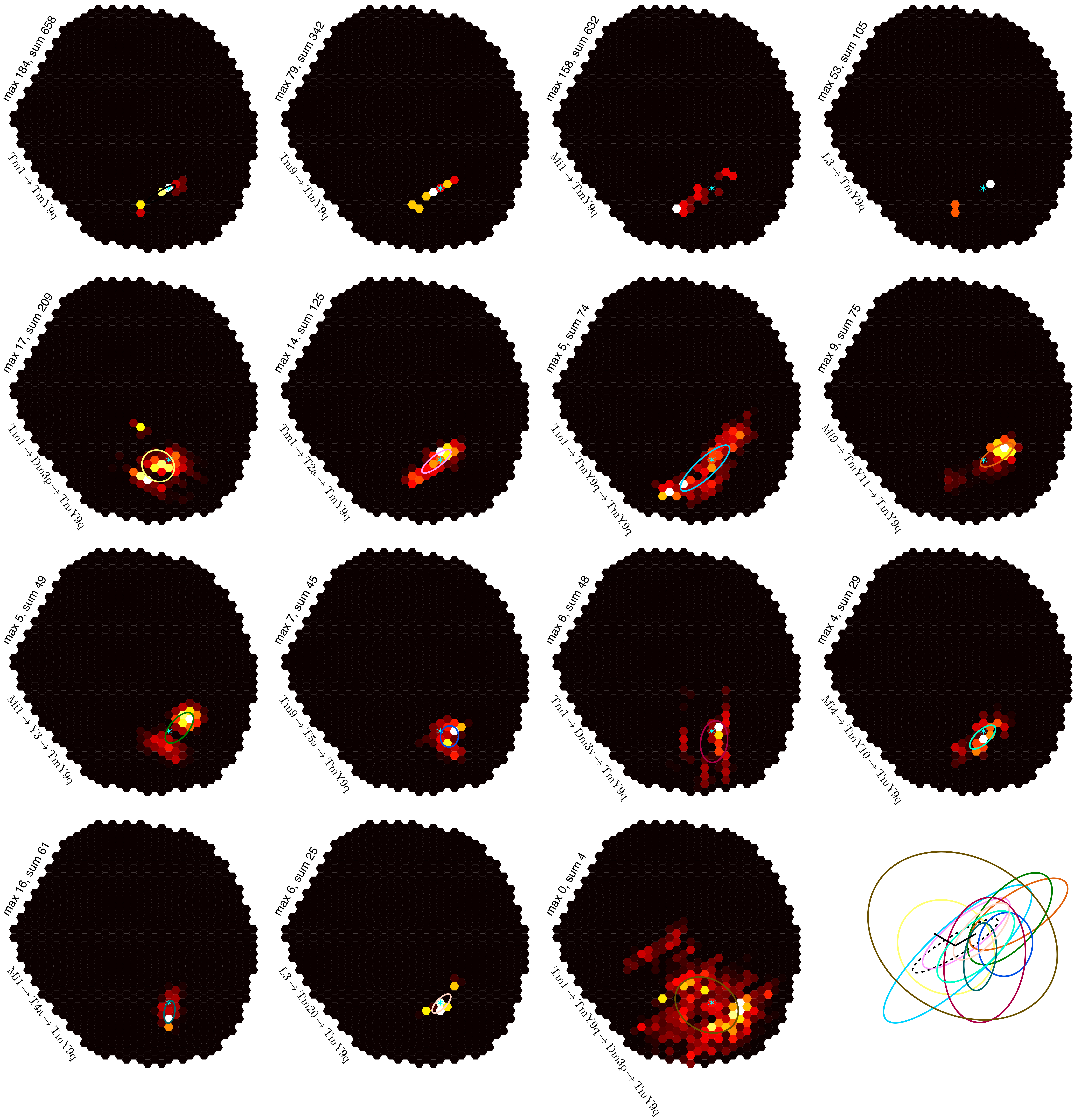

max 184, sum 658
Tm1 → TmY9q
max 79, sum 342
Tm9 → TmY9q
max 158, sum 632
Mi1 → TmY9q
max 53, sum 105
L3 → TmY9q
max 17, sum 209
Tm1 → Dm3p → TmY9q
max 14, sum 125
Tm1 → T2a → TmY9q
max 5, sum 74
Tm1 → TmY9q → TmY9q
max 9, sum 75
Mi9 → TmY11 → TmY9q
max 5, sum 49
Mi1 → Y3 → TmY9q
max 7, sum 45
Tm9 → T5a → TmY9q
max 6, sum 48
Tm1 → Dm3v → TmY9q
max 4, sum 29
Mi4 → TmY10 → TmY9q
max 16, sum 61
Mi1 → T4a → TmY9q
max 6, sum 25
L3 → Tm20 → TmY9q
max 0, sum 4
Tm1 → TmY9q → Dm3p → TmY9q
